# Annotation of biologically relevant ligands in UniProtKB using ChEBI

**DOI:** 10.1093/bioinformatics/btac793

**Published:** 2022-12-09

**Authors:** Elisabeth Coudert, Sebastien Gehant, Edouard de Castro, Monica Pozzato, Delphine Baratin, Teresa Neto, Christian J A Sigrist, Nicole Redaschi, Alan Bridge, The UniProt Consortium, Alan J Bridge, Lucila Aimo, Ghislaine Argoud-Puy, Andrea H Auchincloss, Kristian B Axelsen, Parit Bansal, Delphine Baratin, Teresa M Batista Neto, Marie-Claude Blatter, Jerven T Bolleman, Emmanuel Boutet, Lionel Breuza, Blanca Cabrera Gil, Cristina Casals-Casas, Kamal Chikh Echioukh, Elisabeth Coudert, Beatrice Cuche, Edouard de Castro, Anne Estreicher, Maria L Famiglietti, Marc Feuermann, Elisabeth Gasteiger, Pascale Gaudet, Sebastien Gehant, Vivienne Gerritsen, Arnaud Gos, Nadine Gruaz, Chantal Hulo, Nevila Hyka-Nouspikel, Florence Jungo, Arnaud Kerhornou, Philippe Le Mercier, Damien Lieberherr, Patrick Masson, Anne Morgat, Venkatesh Muthukrishnan, Salvo Paesano, Ivo Pedruzzi, Sandrine Pilbout, Lucille Pourcel, Sylvain Poux, Monica Pozzato, Manuela Pruess, Nicole Redaschi, Catherine Rivoire, Christian J A Sigrist, Karin Sonesson, Shyamala Sundaram, Alex Bateman, Maria-Jesus Martin, Sandra Orchard, Michele Magrane, Shadab Ahmad, Emanuele Alpi, Emily H Bowler-Barnett, Ramona Britto, Hema Bye- A-Jee, Austra Cukura, Paul Denny, Tunca Dogan, ThankGod Ebenezer, Jun Fan, Penelope Garmiri, Leonardo Jose da Costa Gonzales, Emma Hatton-Ellis, Abdulrahman Hussein, Alexandr Ignatchenko, Giuseppe Insana, Rizwan Ishtiaq, Vishal Joshi, Dushyanth Jyothi, Swaathi Kandasaamy, Antonia Lock, Aurelien Luciani, Marija Lugaric, Jie Luo, Yvonne Lussi, Alistair MacDougall, Fabio Madeira, Mahdi Mahmoudy, Alok Mishra, Katie Moulang, Andrew Nightingale, Sangya Pundir, Guoying Qi, Shriya Raj, Pedro Raposo, Daniel L Rice, Rabie Saidi, Rafael Santos, Elena Speretta, James Stephenson, Prabhat Totoo, Edward Turner, Nidhi Tyagi, Preethi Vasudev, Kate Warner, Xavier Watkins, Rossana Zaru, Hermann Zellner, Cathy H Wu, Cecilia N Arighi, Leslie Arminski, Chuming Chen, Yongxing Chen, Hongzhan Huang, Kati Laiho, Peter McGarvey, Darren A Natale, Karen Ross, C R Vinayaka, Qinghua Wang, Yuqi Wang

**Affiliations:** Swiss-Prot Group, SIB Swiss Institute of Bioinformatics, Centre Medical Universitaire, 1211 Geneva 4, Switzerland; Swiss-Prot Group, SIB Swiss Institute of Bioinformatics, Centre Medical Universitaire, 1211 Geneva 4, Switzerland; Swiss-Prot Group, SIB Swiss Institute of Bioinformatics, Centre Medical Universitaire, 1211 Geneva 4, Switzerland; Swiss-Prot Group, SIB Swiss Institute of Bioinformatics, Centre Medical Universitaire, 1211 Geneva 4, Switzerland; Swiss-Prot Group, SIB Swiss Institute of Bioinformatics, Centre Medical Universitaire, 1211 Geneva 4, Switzerland; Swiss-Prot Group, SIB Swiss Institute of Bioinformatics, Centre Medical Universitaire, 1211 Geneva 4, Switzerland; Swiss-Prot Group, SIB Swiss Institute of Bioinformatics, Centre Medical Universitaire, 1211 Geneva 4, Switzerland; Swiss-Prot Group, SIB Swiss Institute of Bioinformatics, Centre Medical Universitaire, 1211 Geneva 4, Switzerland; Swiss-Prot Group, SIB Swiss Institute of Bioinformatics, Centre Medical Universitaire, 1211 Geneva 4, Switzerland; Swiss-Prot Group, SIB Swiss Institute of Bioinformatics, Centre Medical Universitaire, 1211 Geneva 4, Switzerland; European Molecular Biology Laboratory—European Bioinformatics Institute (EMBL-EBI), Hinxton, Cambridgeshire CB10 1SD, UK; Protein Information Resource, University of Delaware, Newark, DE 19711, USA; Protein Information Resource, Georgetown University Medical Center, Washington, DC 20007, USA

## Abstract

**Motivation:**

To provide high quality, computationally tractable annotation of binding sites for biologically relevant (cognate) ligands in UniProtKB using the chemical ontology ChEBI (Chemical Entities of Biological Interest), to better support efforts to study and predict functionally relevant interactions between protein sequences and structures and small molecule ligands.

**Results:**

We structured the data model for cognate ligand binding site annotations in UniProtKB and performed a complete reannotation of all cognate ligand binding sites using stable unique identifiers from ChEBI, which we now use as the reference vocabulary for all such annotations. We developed improved search and query facilities for cognate ligands in the UniProt website, REST API and SPARQL endpoint that leverage the chemical structure data, nomenclature and classification that ChEBI provides.

**Availability and implementation:**

Binding site annotations for cognate ligands described using ChEBI are available for UniProtKB protein sequence records in several formats (text, XML and RDF) and are freely available to query and download through the UniProt website (www.uniprot.org), REST API (www.uniprot.org/help/api), SPARQL endpoint (sparql.uniprot.org/) and FTP site (https://ftp.uniprot.org/pub/databases/uniprot/).

**Supplementary information:**

Supplementary data are available at *Bioinformatics* online.

## 1 Introduction

The UniProt Knowledgebase (UniProtKB, at www.uniprot.org) is a reference resource of protein sequences and functional annotation that covers proteins from all branches of the tree of life (The UniProt Consortium, 2021). UniProtKB includes an expert-curated core of around 568 000 reviewed UniProtKB/Swiss-Prot protein sequence entries and over 229 million unreviewed UniProtKB/TrEMBL entries that are annotated by automatic systems (MacDougall *et al.*, 2020) (statistics for release 2022_04 of October 2022). UniProtKB provides a wealth of information on protein sequences and their functions, including the binding sites of biologically relevant or ‘cognate’ ligands (the term used in the remainder of this article) (Das and Orengo, 2018; Tyzack *et al.*, 2018) such as activators, inhibitors, cofactors and substrates, which are crucial to protein function. UniProt curators capture this knowledge through expert literature curation and from experimentally resolved protein structures in the protein data bank (PDB/PDBe) (Armstrong *et al.*, 2020; Burley *et al.*, 2021; Velankar *et al.*, 2021), removing adventitious ligands that are technical artefacts and mapping experimentally observed ligands in PDB to their cognate equivalents by reference to a curated list of known cognate ligands.

Here, we describe improvements to the annotation of cognate ligands and their binding sites in UniProtKB using the chemical ontology ChEBI (Chemical Entities of Biological Interest, www.ebi.ac.uk/chebi/) (Hastings *et al.*, 2016). We have performed a complete reannotation of cognate ligands and their binding sites in UniProtKB, replacing textual descriptions of ligands with stable unique identifiers from the ChEBI ontology, and now use ChEBI as the reference vocabulary for all new cognate ligand binding site annotations. This work makes knowledge of cognate ligands and their binding sites in UniProtKB easier to find and access. It provides improved support for the design of biochemical experiments (Fleischhacker *et al.*, 2015; Frederick *et al.*, 2022) and computational approaches (Das *et al.*, 2021; Littmann *et al.*, 2021; Wehrspan *et al.*, 2022; Wu *et al.*, 2018) to elucidate protein functions and interactions, and enhances interoperability with other resources providing knowledge of cognate ligands such as PDBe (Mukhopadhyay *et al.*, 2019), BioLiP (Yang *et al.*, 2013), FireDB (Maietta *et al.*, 2014), MetalPDB (Putignano *et al.*, 2018) and PDBBind (Liu *et al.*, 2015).

## 2 Materials and methods

### 2.1 Changes to the UniProt data model and formats

Most sequence annotations (also called ‘features’) in UniProtKB, including the cognate ligand binding site annotations that are the subject of this work, consist of three main elements. The ‘feature location’ defines the sequence region or amino acid residue position that is annotated, the ‘feature key’ specifies the type of each feature, and the ‘feature description’ provides a textual description, which for cognate ligands includes the name of the ligand and other relevant information, such as numbering (of multiple ligands of the same type) and ligand roles. We structured this description for binding site annotations into several fields (described in the online documentation at www.uniprot.org/release-notes/2022-08-03-release), to standardize the description of a ligand, and optionally the bound part of the ligand (such as the iron atom in a heme, or a nucleotide in a macromolecule such as DNA), with the ChEBI ontology. We illustrate this new data model with examples in Section 3. We also simplified the range of feature keys that are used for cognate ligand binding site annotations, which prior to this work were the following:

- ‘CA_BIND’, which denotes a sequence region that binds to calcium;
- ‘METAL’, which denotes a sequence position that binds a metal;
- ‘NP_BIND’, which denotes a sequence region that binds a nucleotide phosphate;
- ‘BINDING’, which denotes a sequence position that binds any type of chemical entity;
- ‘REGION’, which denotes a sequence region of interest in a protein (including a region that binds a ligand).

The ChEBI ontology provides a means to search for any ligand or class of ligand represented in ChEBI, at any desired level of specificity, without requiring ligand-specific feature keys. We therefore deprecated the feature keys ‘CA_BIND’, ‘METAL’ and ‘NP_BIND’, and now use the feature key ‘BINDING’ for all binding site annotations for all cognate ligands. We also recurated all cognate ligand binding sites of interest found in features of the type ‘REGION’ and moved them to ‘BINDING’. Finally, we modified all UniRules (MacDougall *et al.*, 2020), including HAMAP (Pedruzzi *et al.*, 2015) and PROSITE (Sigrist *et al.*, 2013) rules, to provide binding site annotations using ChEBI identifiers in the new data model described here.

### 2.2 Mapping of legacy text annotations of cognate ligand binding sites in UniProtKB to ChEBI

UniProtKB previously described cognate ligands in binding site annotations using text labels, such as ‘lipid’, ‘cholesterol’, ‘heme’, ‘heme b’, ‘divalent metal’ or ‘zinc’. To standardize the descriptions of biologically relevant ligands in binding site annotations in UniProtKB, we created a one-to-one mapping between each such text label and the corresponding ChEBI identifier and used that mapping to reannotate all legacy data.

We extracted unique ligand descriptions from binding site annotations linked to each of the feature keys ‘CA_BIND’, ‘METAL’, ‘NP_BIND’ and ‘BINDING’, as well as ‘REGION’ annotations with the word ‘binding’ in the feature description, and mapped each of the text labels found to the corresponding ChEBI identifier manually. During the mapping, we selected the ChEBI that represents the major microspecies of the ligand (the predominant protonation state) at pH 7.3, which is the convention used in UniProtKB and the Rhea reaction knowledgebase (www.rhea-db.org) (Bansal *et al.*, 2022). If an appropriate ChEBI entity was not already available, then we submitted the required structure to ChEBI for inclusion in the chemical ontology. We also assigned a ‘UniProt name’ to each ChEBI entity used in our annotations, which as its name suggests, is a specific synonym that is created and used by UniProt (and is also used in Rhea).

Some ligand text labels presented with multiple possible mappings to ChEBI—sometimes due to stereochemistry issues—while some described generic classes of chemicals or roles such as cofactor, hormone or odorant, which we could not map to any defined structure. We examined each of these cases in turn and, where necessary, recurated them, using information from the literature, the PDB and the UniProtKB protein sequence records concerned, including existing Rhea reaction annotations, before selecting the most appropriate mapping to ChEBI. In total, we recurated over 100 such ambiguous ligands.

Once complete, we used the mapping of defined cognate ligands to replace legacy text labels in UniProtKB with the corresponding identifiers from ChEBI. We also used additional information from the existing annotations, such as ligand numbering and roles, to populate the corresponding data fields in the new structured data model.

We did not yet systematically recurate binding site annotations for enzymes in UniProtKB with the generic text label ‘substrate’, which does not specify which of the possible substrate(s) are bound. We are continuing to map these legacy ‘substrate’ annotations to specific ChEBI identifiers, using Rhea annotations and other information such as ligand data from PDBe records where available, mapped to UniProt sequences using the SIFTS framework (Dana *et al.*, 2019).

### 2.3 UniProt tools and services to exploit ligand binding site annotations

We modified the UniProt website www.uniprot.org, UniProt REST API www.uniprot.org/help/api and UniProt SPARQL endpoint sparql.uniprot.org/, to support searches for ligand binding site annotations using ChEBI identifiers, ligand names, synonyms and chemical structures from ChEBI encoded as InChIKeys. The InChIKey is a simple hash representation of chemical structures that provides a convenient means to search and map chemical structure databases (see www.inchi-trust.org/).

## 3 Results

### 3.1 Structuring cognate ligand binding site annotations in UniProtKB using ChEBI

The annotation of cognate ligand binding sites in UniProtKB using the chemical ontology ChEBI was made available from UniProt release 2022_03 of August 2022. This initial release featured 776 unique ligands from ChEBI, which were involved in over 980 000 binding site annotations for over 200 000 UniProtKB/Swiss-Prot protein sequence records, and over 65 million binding site annotations for over 17 million protein sequence records for the whole of UniProtKB, including UniProtKB/TrEMBL. We provide a complete list of all cognate ligands used in binding site annotations in UniProtKB release 2022_03 in Supplementary Table S1. This list is part of a larger set of allowed ligands for binding site annotations in UniProtKB, which also includes all ChEBI entities used in Rhea reactions.

The new data model improves the consistency of annotations while retaining flexibility. It supports the annotation of binding sites for ligands described at any level of granularity in ChEBI, from broad classes of ligands such as ‘metal cation’ (CHEBI: 25213) or ‘heme’ (CHEBI: 30413), to structurally defined ligands such as ‘Fe(2+)’ (CHEBI: 29033) or ‘heme b’ (CHEBI: 60344). It also supports the annotation of binding sites for ligands that are parts of larger macromolecules. The example below shows one such case, where amino acid 146 of yeast L-lactate dehydrogenase (UniProtKB/Swiss-Prot entry P00175) binds to the iron atom (CHEBI: 18248) of heme b (CHEBI: 60344) (this form of heme b represents the predominant protonation state at pH7.3, the form chosen by convention in UniProtKB). The ‘evidence’ field lists the evidences that support the annotation. Each evidence is described by a term from the Evidence and Conclusions Ontology ECO (Nadendla *et al.*, 2022), and the source of the information, here experiments published in two peer-reviewed articles (Cunane *et al.*, 2002; Xia and Mathews, 1990) and protein structures 1FCB and 1KBI from the PDB.

**Table:** 

FT	BINDING	146
FT		/ligand=‘heme b’
FT		/ligand_id=‘ChEBI:CHEBI:60344’
FT		/ligand_part=‘Fe’
FT		/ligand_part_id=‘ChEBI:CHEBI:18248’
FT		/note=‘axial binding residue’
FT		/evidence=‘ECO:0000269|PubMed:11914072,
FT		ECO:0000269|PubMed:2329585,
FT		ECO:0007744|PDB:1FCB,
FT		ECO:0007744|PDB:1KBI’

We refer readers to the online documentation at www.uniprot.org/release-notes/2022-08-03-release, which provides additional examples of binding site annotations in the UniProtKB formats text, XML and RDF/XML.

### 3.2 UniProt tools and services to access and query cognate ligand binding site annotations made with ChEBI

Users can access and query UniProtKB cognate ligand binding site annotations made with ChEBI using the UniProt website, REST API and SPARQL endpoint.

#### 3.2.1 UniProt website

**Fig. 1.**
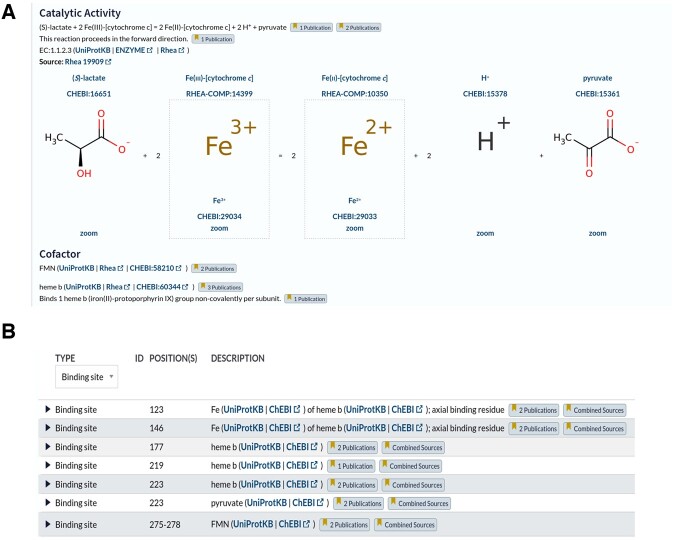
Website view of small molecule annotations in UniProtKB, including cognate ligand binding site annotations—from www.uniprot.org/uniprotkb/P00175/entry. All small molecule annotations are shown in the ‘Function’ section. (**A**) The ‘Catalytic Activity’ subsection describes enzymatic reactions using Rhea (which is based on ChEBI), while cofactors are described using ChEBI in the ‘Cofactor’ subsection. Standardization of reaction and cofactor descriptions was performed in previous work, and is shown here for completeness. (**B**) The ‘Features’ subsection displays the available binding site annotations for cognate ligands described using ChEBI, the subject of this work. Each ligand has a link ‘UniProtKB’ to launch searches for other proteins binding this ligand, a link out to ‘ChEBI’, and expandable sections like ‘Publications’ to examine provenance and evidence

The UniProt website www.uniprot.org provides access to UniProtKB protein sequence records and annotations, including cognate ligand binding site annotations for each protein (Fig. 1). Users can now query the website for proteins that bind cognate ligands of interest using identifiers, names, synonyms and chemical structures (encoded as InChIKeys) from ChEBI using the advanced query builder. The complete ChEBI ontology is indexed, so that searches using identifiers for higher-level grouping classes in the ChEBI ontology will retrieve UniProtKB records with binding site annotations to all child classes. ChEBI identifiers entered by users are automatically mapped to those of the major microspecies at pH 7.3, which is the form used in UniProtKB and Rhea, using a mapping file provided by Rhea.

The sample query shown below will retrieve all proteins with binding site annotations for any kind of heme, using the ChEBI identifier for that grouping class, which is ChEBI: 30413:

1. (ft_binding:‘CHEBI: 30413’)The result set will include all proteins with binding site annotations for any form of heme (CHEBI: 30413), including ‘heme b’ (CHEBI: 60344), ‘heme c’ (CHEBI: 61717) and all others. To retrieve proteins with binding site annotations for specific forms of heme, users can simply change the ChEBI identifier to that of the form desired, here ChEBI: 60344 for ‘heme b’:
2. (ft_binding: ‘CHEBI: 60344’)Users can also perform searches for specific ligands using the chemical structure represented as an InChIKey, if using a chemical structure database other than ChEBI:
3. (ft_binding: KABFMIBPWCXCRK-RGGAHWMASA-J)Users can elect to ignore the charge, by removing the third block of the InChIKey, as in this example:
4. (ft_binding: KABFMIBPWCXCRK-RGGAHWMASA)They may also elect to ignore both stereochemistry and charge, by removing both the second and third blocks of the InChIKey:
5. (ft_binding: KABFMIBPWCXCRK)Users can also combine searches for cognate ligand binding site annotations with other types of annotations, as in this query for human mitochondrial flavoproteins (i.e. proteins with annotated binding sites for some CHEBI: 30527 - flavin) that are linked to genetic diseases defined by the resource Online Mendelian Inheritance in Man (Hamosh *et al.*, 2021):
6. (ft_binding: ‘CHEBI: 30527’) AND (cc_scl_term: SL-0173) AND (organism_id: 9606) AND (cc_disease:*)

We provide complete documentation on searching for small molecule data in UniProtKB, including ligands described in binding site annotations, at www.uniprot.org/help/chemical_data_search.

#### 3.2.2 UniProt REST API

The UniProt REST API (www.uniprot.org/help/api) allows users to query and process UniProt data programmatically and to specify the required output format for query results (such as txt, xml, rdf, tsv, etc.) and, for the tab-separated format, the desired annotation fields. The simplest way to create URLs for programmatic use is by using the advanced query builder to set the desired query fields and values, perform the search and click the ‘Download’ button, which opens a panel with a ‘Generate URL for API’ link. Users can now query the UniProt REST API with identifiers, names, synonyms and chemical structures from ChEBI for ligand-binding site annotations.

#### 3.2.3 UniProt SPARQL endpoint

The UniProt SPARQL endpoint sparql.uniprot.org allows users to query UniProt RDF data and RDF data from other SPARQL endpoints using federated SPARQL queries. It now supports queries for ligand-binding site annotations using identifiers, names, synonyms and chemical structure data from ChEBI. We demonstrate this capability using a federated SPARQL query that combines the UniProt SPARQL endpoint and that of the Integrated Database of Small Molecules (IDSM) (Galgonek and Vondrášek, 2021; Kratochvil *et al.*, 2019). IDSM supports fingerprint-guided chemical similarity and substructure searches in a number of chemical datasets, including ChEBI, using Sachem, a high-performance open source chemical cartridge (Kratochvil *et al.*, 2018). This federated SPARQL query allows UniProt to borrow that functionality from IDSM; it will find all proteins that bind to ligands with structures similar to that of a query ligand, in this case, heme b (specified using SMILES or Simplified Molecular-Input Line-Entry notation) (http://opensmiles.org). The UniProt SPARQL endpoint queries that of IDSM, which returns the set of chemical entities in ChEBI that are similar to the query ligand heme b (above a Tanimoto similarity score threshold of 0.8) and then searches for proteins in UniProtKB with binding site annotations for those ligands, which it then returns to the user.

SELECT? uniprot? mnemonic? proteinName? ligandSimilarityScore? ligand

WHERE {

 SERVICE <https://idsm.elixir-czech.cz/sparql/endpoint/chebi> {

  [sachem: compound? ligand; sachem: score? ligandSimilarityScore]

  sachem: similaritySearch

   [

   sachem: query

     ‘CC1 = C(CCC([O-])=O)C2=[N+]3C1 = Cc1c(C)c(C =  C)c4C=C5C(C)=C(C = C)C6=[N+]5[Fe-]3(n14)n1c(=C6)c(C)c(CCC([O-])=O)c1 = C2’;

   sachem: cutoff ‘8e-1’ ^  ^ xsd: double;

   sachem: aromaticityMode sachem: aromaticityDetect;

   sachem: similarityRadius 1;

   sachem: tautomerMode sachem: ignoreTautomers

  ]

 }

 ? uniprot up: mnemonic? mnemonic.

 ? uniprot up: recommendedName/up: fullName? proteinName.

 ? uniprot up: annotation? annotation.

 ? annotation a up: Binding_Site_Annotation;

 up: ligand/rdfs: subClassOf? ligand.

}

ORDER BY DESC(? ligandSimilarityScore)

This type of query could be useful in the study of 3D protein structures and protein structure models. Given the SMILES representation of a non-cognate ligand from an experimentally determined 3D protein structure from PDBe, users can retrieve similar cognate ligands from UniProtKB that could replace it to create a more biologically relevant structure. Predicted 3D protein structure models from state-of-the-art methods such as AlphaFold (Jumper *et al.*, 2021; Varadi *et al.*, 2022) lack ligands altogether, and methods that transfer experimental ligands from similar structures in PDBe (Hekkelman *et al.*, 2022) might exploit UniProtKB as a source of cognate ligands for this transfer. We provide more sample queries in the online documentation for the UniProt SPARQL endpoint at https://sparql.uniprot.org/.well-known/sparql-examples/, while the developers of the IDSM SPARQL endpoint provide additional documentation at https://idsm.elixir-czech.cz/sparql/doc/manual.html.

## 4 Conclusions and future work

We have structured and reannotated cognate ligand binding sites in UniProtKB using ChEBI and report new tools and services to exploit this improved ligand dataset via the UniProt website and APIs. This work is part of an ongoing program to standardize all small molecule annotations in UniProtKB using ChEBI and builds on previous improvements to the annotation of enzymes and transporters in UniProtKB using the Rhea knowledgebase of biochemical reactions, which uses ChEBI to represent reactants (Bansal *et al.*, 2022; Morgat *et al.*, 2020). We continue to work to improve the UniProtKB cognate ligand dataset through expert literature curation, supported by machine learning approaches to target relevant literature for information extraction (Allot *et al.*, 2021; Islamaj *et al.*, 2021), and through the development of improved pipelines for the import and curation of ligand data from PDBe.

## Supplementary Material

btac793_Supplementary_Data
